# Foley catheter with noble metal alloy coating for preventing catheter-associated urinary tract infections: a large, multi-center clinical trial

**DOI:** 10.1186/s13756-021-00907-w

**Published:** 2021-02-25

**Authors:** Ylva Kai-Larsen, Stefan Grass, Bhaumik Mody, Swati Upadhyay, Hargovind L. Trivedi, Dilip. K. Pal, Santosh Babu, Bikash Bawari, Shrawan. K. Singh

**Affiliations:** 1 Bactiguard AB, Alfred Nobels Allé 150, 146 48 Tullinge, Stockholm, Sweden; 2 Ethitrials Clinical Research Solution, Navrangpura, Ahmedabad India; 3 Apollo Hospitals International Limited, GIDC Estate, Bhat, India; 4 Institute of Kidney Diseases and Research Centre (IKDRC), Institute of Transplantation Sciences (ITS), B. J. Medical College and Civil Hospital Campus, Ahmedabad, Gujarat India; 5 Institute of Post Graduate Medical Education and Research, Bose Road, Kolkata, India; 6 Department of General Surgery, Gandhi Hospital, Musheerabad, Secunderabad, Telangana India; 7 Marwari Hospital and Research Centre S.J. Road, Athagaon, Assam India; 8 Department of Urology, Postgraduate Institute of Medical Education and Research, Chandigarh, India

**Keywords:** Catheter, Catheter-associated urinary tract infection, Foley, BIP foley catheter, Noble metal alloy, Coating, Infection control

## Abstract

**Background:**

Catheter-associated urinary tract infections (CAUTI) are among the most frequent healthcare-associated infections in the world. They are associated with increased mortality, prolonged hospital stay and increased healthcare costs. The objective of this study was to evaluate the efficacy of the noble metal alloy (NMA) coated BIP Foley Catheter in preventing the incidence of symptomatic CAUTI in a large cohort of patients in India.

**Methods:**

This multi-center, prospective study included 1000 adult patients admitted to six hospitals across India for urology, surgery and ICU requiring urethral catheterization and admission for ≥ 48 h. Patients were allocated to the NMA-coated BIP Foley Catheter group or a non-coated control catheter group, with a randomization ratio of 3:1. CAUTI surveillance was conducted at study entry, upon catheter removal, and 2 days after catheter removal. For statistical analysis, categorical data (e.g. gender) were compared using the chi-square or Fischer test, and numerical data were compared using the two-sample *t*-test. Associations were evaluated using logistic regression.

**Results and conclusions:**

The incidence of symptomatic CAUTI was reduced by 69% in the BIP Foley Catheter group compared to the control group (6.5 vs 20.8 CAUTI/1000 catheter days), with an incidence rate ratio of 0.31 (95% confidence interval: 0.21–0.46; *p* < 0.001). A reduction in the cumulative CAUTI incidence was evident in the BIP Foley Catheter group within 3 days after catheterization; this reduction was maintained up to ~ 30 days, and the largest reductions were seen between 3 and 11 days. There were no serious adverse events related to either catheter, and the percentage of patients with ≥ 1 adverse event was significantly lower in the NMA-coated BIP Foley Catheter group than in the control group (21.6% vs. 48.4%; *p* = 0.001). In conclusion, the NMA-coated BIP Foley Catheter was effective in reducing CAUTI and was well tolerated, with a lower incidence of adverse events compared to the uncoated catheter.

*Trial registration* This study was registered prospectively (28 September 2015) in the Clinical Trials Registry of India (trial number CTRI/2015/09/006220; http://ctri.nic.in/Clinicaltrials/showallp.php?mid1=12631&EncHid=&userName=bactiguard).

## Introduction

Healthcare-associated infections (HAI) are common and represent a significant global burden of disease, with increased mortality, prolonged hospital stay and increased healthcare costs [1–3]. The use of invasive devices, such as urinary catheters, central lines and ventilators, is associated with a high frequency of HAI. Since urinary catheters are the most commonly used medical device in the world, catheter-associated urinary tract infections (CAUTI) are among the most frequent HAI, with more than 150 million cases/year [4]. Patients with CAUTI have prolonged hospital stays and additional costs ranging between $876 and $10,197 are incurred [5–7]. CAUTI are often caused by multidrug-resistant strains of bacteria, which are a global threat to human health [8]. In India, the incidence of CAUTI varies between regions and hospitals, with intensive care unit (ICU) rates of 4.4 CAUTI/1000 catheter days and ward rates of 18 CAUTI/1000 catheter days having been reported [9].

According to the US Centers for Disease Control and Prevention (CDC), CAUTI are defined by a positive urine culture (bacteriuria) together with at least one symptom [10]. Bacteriuria occurs with a frequency of 3–6%/day in catheterized patients [11], and, as well as the chance of causing symptoms, there are risks of secondary bloodstream infection (bacteremia) and urosepsis (2% of CAUTI cases) [12].

Risk factors for CAUTI include older age, female gender, diabetes mellitus, and extended duration of catheterization. Many CAUTI are attributable to contamination of the catheter, either during insertion or during use, when the drainage system may serve as a source of contamination [13].

Evidence-based prevention strategies have been introduced to reduce the risk of CAUTI, and it is now standard practice to ensure sterility at the time of insertion, to use closed drainage systems, and to minimize the duration of catheterization [14]. In addition, catheters coated with antiseptic and antimicrobial compounds such as silver ions, antibiotics, and noble metal alloys (NMAs) have been developed to reduce the risk of bacterial colonization [15–17]. One NMA-coated latex catheter (Bactiguard infection protection [BIP] Foley) has a non-releasing coating of gold, silver, and palladium, and has been shown to reduce the incidence of CAUTI in several settings (e.g. ICU, burn units, rehabilitation) [18–23]. However, a part of the available data are from small, non-randomized or retrospective studies performed without adherence to an accepted definition of CAUTI.

We performed a study to assess the efficacy of the NMA-coated BIP Foley Catheter in reducing the risk of CAUTI among patients in India. The intention was for this to be the largest randomized controlled trial of this device, with adequate duration of catheterization (≥ 2 days) and latency period post-catheterization, as well as adherence to an accepted definition of CAUTI (i.e. that of the CDC).

## Methods

### Design and participants

This was a prospective, multi-center, randomized, controlled study. Adults aged > 18 years requiring urethral catheterization (closed drainage system) for ≥ 48 h were eligible for enrolment, provided they were being admitted to hospital for urology, surgery and an ICU stay. Candidate patients were excluded if they were pregnant or breastfeeding, were receiving antibiotic treatment for a UTI or catheterization, had a latex allergy, or had undergone previous urinary tract surgery likely to interfere with the study results. The study was performed as part of a post-marketing commitment in India, agreed between Bactiguard AB (manufacturer of the BIP Foley Catheter) and the Center for Medical Device Evaluation at the Central Drugs Standard Control Organization. The study was performed in six hospitals spanning multiple regions of India; (1) Apollo Hospitals International Limited (West), (2) Civil Hospital Campus, Ahmedabad (West)- (3) Chandigarh Hospital (North), (4) Kolkata Hospital (East), (5) Gandhi Hospital, Hyderabad (South) and (6) Marwari Hospital, Guwahati (North-East). Approval of the study protocol was obtained from the Institutional Review Board (i.e. ethical committee) at each participating hospital. The study was conducted according to the declaration of Helsinki (6th revision 2008), ISO 14155, and Good Clinical Practice clinical research requirements in India (CDCSO 2004).

### Randomization

A total of 1000 patients were randomly allocated in a 3:1 ratio to two groups, one using the NMA-coated BIP Foley Catheter (Bactiguard® Infection Protection, Bactiguard AB, Tullinge, Sweden) and the other using an uncoated latex control catheter (Bardia; Bard / Becton Dickinson, USA). In total 1003 patients fulfilled eligibility for participation in the study. However, two patients declined participation before study start and one patient was excluded due to delayed surgery and thus no need for catheterization.

Block randomization of eight patients per block were used in 3:1 ratio, and no special adjustments or further stratification based on any patient characteristics were performed. The randomization code was generated using Statistical Analysis Software (SAS), Version 9.3 (SAS Institute Inc., India). The laboratory evaluation testing was blinded, but study patients and healthcare professionals treating them were unblinded.

### Procedures

**Fig. 1:**
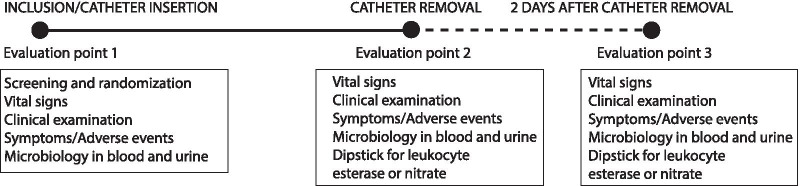
Study design. Three evaluation points were included: inclusion into the study, catheter removal and 2 days after catheter removal

The study included three evaluation timepoints: inclusion into the study, catheter removal and 2 days after catheter removal (Fig. 1). Catheters were removed according to guidelines/recommendations applicable to the patient’s clinical indication [13]. Urine samples were assessed by a dipstick test, a pyuria test, and a urine culture test (standard microbiological testing). Patient safety assessments included vital signs, physical examination and laboratory tests performed according to standard hospital routines (Fig. 1).

### Endpoints

**Fig. 2:**
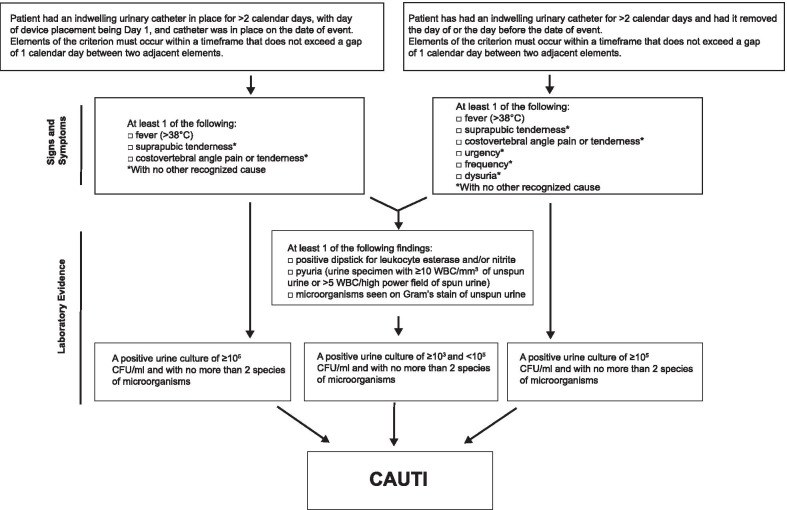
Definition of catheter–associated urinary tract infection (Centers for Disease Control and Prevention, National Healthcare Safety Network, 2014)

The primary endpoint was the incidence of symptomatic CAUTI, defined according to CDC criteria (2014). These criteria included: onset of infection within 2 days after removal of catheter; catheterization time ≥ 2 days; bacterial growth of ≥10^5^ or 10^3^–10^5^ CFU/ml in a urine specimen; a positive result from one of the following tests: dipstick for leukocyte-esterase or nitrate; Gram stain; or pyuria and at least one sign/symptom of CAUTI (Fig. 2). Secondary outcomes included numbers of spontaneous urinary and blood cultures, duration of hospitalization, and bacteriuria. Data were collected using electronic case report forms (eCRFs) and audited to ensure consistency. No significant protocol violations were observed.

### Statistical analysis

The study size (i.e. 1000 patients) was specified by the Center for Medical Device Evaluation within the post-marketing commitment for the BIP Foley Catheter. Consequently, no sample size calculation was performed.

Results were analyzed on an “intention to treat” basis using SAS (Version 9.3) and R (Version 3.6.3) and summarized using standard descriptive statistics. Categorical data (e.g. gender) were compared using the chi-square or Fischer test, and numerical data (e.g. age, height and weight) were compared using the two-sample t-test. Associations were evaluated using logistic regression, with treatment arm as an independent variable and the logarithm of catheter days as an offset. All *p*-values were calculated two-sided and *p*-values less than 0.05 were considered statistically significant.

## Results

**Fig. 3:**
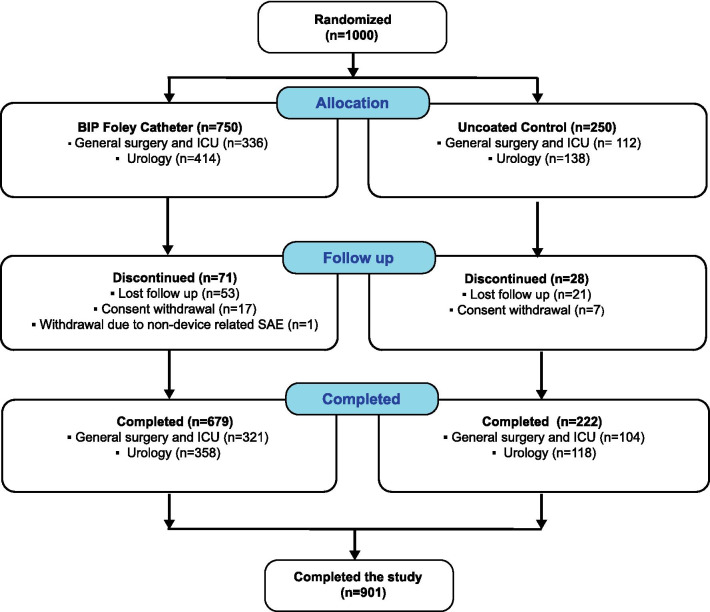
Patient disposition. In total, 1000 patients were randomized and 901 patients completed the study. The distribution of patient types was similar in the two study groups

Patients were enrolled between December 2015 and April 2018; 750 were randomized to receive the BIP Foley Catheter and 250 were allocated to the control group (uncoated catheter). Nine hundred and one patients completed the study per protocol, and 99 patients (71 in the BIP Foley Catheter group and 28 in the control group) were withdrawn early (Fig. 3). Patients’ baseline characteristics (e.g. age, weight, height) were similar in both groups, although there was a small difference in gender distribution: in the BIP Foley Catheter arm, 235 (31%) patients were female, compared with 101 (40%) in the control arm. The mean duration of catheterization, 11 days, was the same in both groups. The total number of catheterization days was 7,987 with the BIP Foley Catheter and 2,551 with the control device. No statistically significant differences were observed in prophylactic antibiotic use (Table 1).

**Table 1:** Baseline demographics and outcomes in the groups receiving noble metal coated catheter or uncoated control catheter

	BIP Foley n = 750	Control n = 250	*p*-value	Relative reduction (RR) (%)
*Demographics and comorbidities*				
Age, mean (SD)	48 (15)	47 (15)	0.0908	NA
Weight, mean (SD)	65 (34)	69 (58)	0.27	NA
Height, mean (SD)	161 (11)	162 (9)	0.39	NA
Sex, female, n (%)	235 (31)	101 (40)	0.0106	NA
Diabetes mellitus	46 (6.1)	19 (7.6)	0.4	NA
Cancer	5 (0.7)	5 (2)	0.07	NA
*Distribution of patients at each site and ward type*				
Site 1 General surgery and ICU	102 (14)	34 (14)	NA	NA
Site 2 Urology	6 (1)	2 (1)	NA	NA
Site 3 Urology	96 (13)	32 (13)	NA	NA
Site 4 Urology	228 (30)	76 (30)	NA	NA
Site 5 General surgery	234 (31)	78 (31)	NA	NA
Site 6 Urology	84 (11)	28 (11)	NA	NA
Total General surgery and ICU	336 (45)	112 (45)	NA	NA
Total Urology	414 (55)	138 (55)	NA	NA
*CAUTI*				
CAUTI incidences (%) TOTAL	52 (6.9)	53 (21.2)	< 0.001	67%
CAUTI/1000 catheter days TOTAL	6.5 (52/(7987 * 10^−3^))	20.8 (53/(2551 days * 10^−3^))	< 0.001	69%
General surgery and ICU (site 1 and 5) CAUTI/1000 catheter days	12.3 (32/(2612 * 10^−3^))	47.9 (37/(773 * 10^−3^))	< 0.001	70%
Urology (site 2–4, 6) CAUTI/1000 catheter days	3.7 (20/5375 * 10^−3^))	9.0 (16/(1778 * 10^−3^))	0.006	58%
*Bacteriuria*				
Positive growth at inclusion TOTAL n (%)	30 (4)	16 (6.4)	0.11	NA
Positive growth TOTAL n (%)	209 (28)	161 (64)	< 0.001	57%
Positive growth, ≥ 10^5^ CFU/ml n (%)	89 (11.9)	67 (26.8)	< 0.001	56%
Positive growth, < 10^5^ ≥ 10^3^, CFU/ml n (%)	66 (8.8)	53 (21.2)	< 0.001	59%
*Antibiotics, catheterization days, hospital*				
Antibiotics prophylaxis*, days/antibiotic treated study patient	8.2	8.5	0.54	No significant difference
Antibiotics prophylaxis, days/all study patients	4.7	5.0	0.47	No significant difference
Duration of catheterization, days	11.1	10.9	0.79	No significant difference
Duration of hospital admission, days	6.5	6.3	0.59	No significant difference
*Adverse events*				
Total number of AE (%)	162 (21.6%)	121 (48.4%)	< 0.001	55%
Total patients with at least one AE n,	112 (14.9%)	80 (32.0%)	< 0.001	53%

*Antibiotics were received for other reasons than catheterization

### CAUTI incidence

**Fig. 4:**
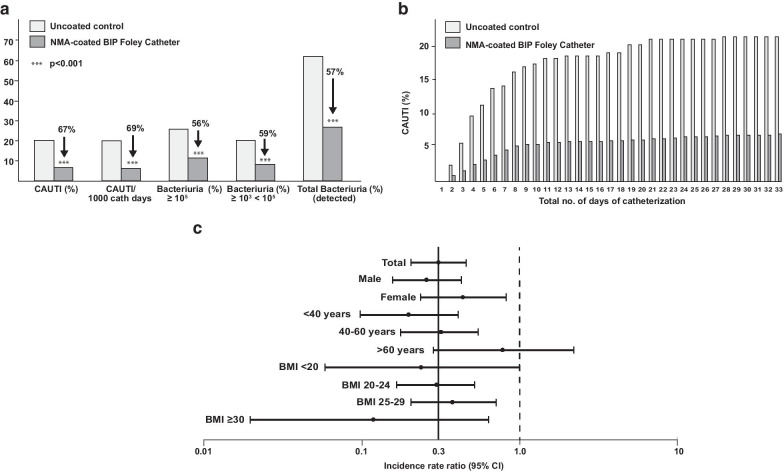
CAUTI incidence rates, for the whole study (**a**), over time (**b**) and in patient subgroups (**c**). Significant reductions were observed with the NMA-coated BIP Foley Catheter compared to the uncoated control catheter

Symptomatic CAUTI occurred during the study period in 52/ 750 patients (6.9%) in the BIP Foley Catheter group, and 53/250 patients (21.2%) in the control group. Thus, the proportion of patients affected by CAUTI was reduced by 67% in the BIP Foley Catheter group (odds ratio [OR] 0.28; 95% confidence interval [CI] 0.18–0.42; *p* < 0.001; Table 1 and Fig. 4a). Similar results were seen when the duration of catheterization was taken into consideration: the incidence rates were 6.5 and 20.8 CAUTI/1000 catheterization days in the two groups respectively, signaling a 69% reduction in CAUTI incidence with the BIP Foley Catheter (incidence rate ratio 0.31; 95% CI 0.21–0.46; *p* < 0.001; Fig. 4a).

Cumulative CAUTI case counts were significantly reduced in the BIP Foley Catheter group versus the control group as early as 3 days post-catheterization (i.e. total number of days of catheterization = 2, see Fig. 4b), and this difference was maintained up to ~ 30 days. The between-group difference in cumulative cases was most pronounced between 3 and 11 days (Fig. 4b).

Subgroup analyses revealed that the BIP Foley Catheter reduced the CAUTI incidence rate regardless of ward type (urology versus surgery and ICU, see Table 1), patients’ gender, age or BMI, although the reduction was not statistically significant in patients aged > 60 years or in those with BMI < 20 (Fig. 4c).

### Microbiology

**Fig. 5:**
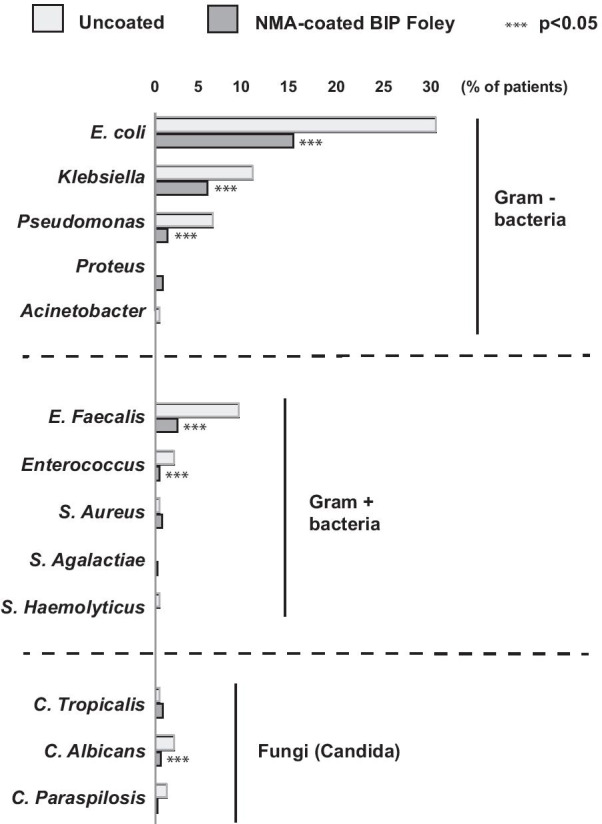
Microbes that caused bacteriuria/CAUTI. Incidence rates for many strains were significantly reduced with the BIP Foley Catheter compared to the uncoated control catheter

A significantly lower proportion of patients in the BIP Foley Catheter group experienced bacteriuria (≥ 10^5^ CFU/ml) than in the control group: 89/750 (12%) versus 67/250 (27%). The relative reduction was 56% (*p* < 0.001). Similar relative reductions of 50–60% were observed when bacteriuria was defined by lower bacterial counts (Fig. 4a). The four most common types of infection-causing organism were *Escherichia coli, Klebsiella* spp.*, Pseudomonas* spp. and *Enterococcus faecalis* in both study arms, and the infection rate for each one was reduced significantly by the BIP Foley Catheter (Fig. 5). The infection rates for *Enterococcus* and the fungi *Candida albicans* were also significantly lower in the BIP Foley Catheter group than in the control group (Fig. 5). There was no spontaneous collection of urine or blood samples for culture testing in either study arm.

### Safety

**Fig. 6:**
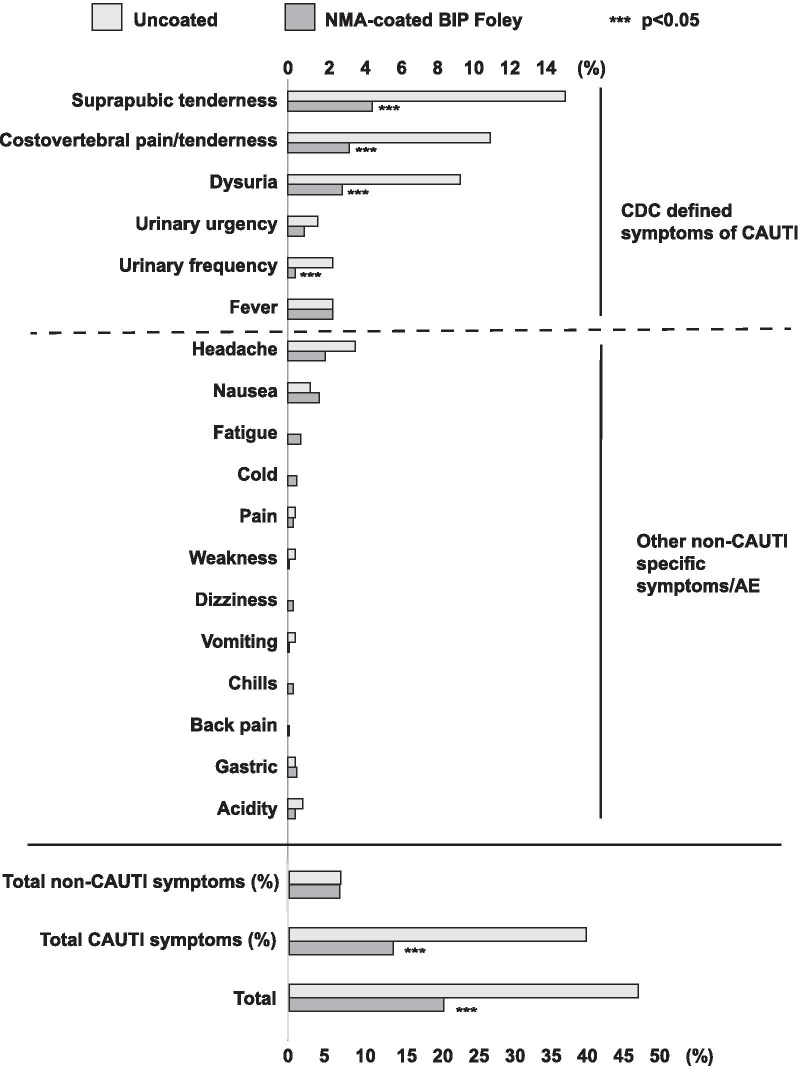
Adverse events. The frequency of total adverse events was significantly lower in the BIP Foley Catheter group than in the control group. There was no significant between-group difference in AEs unrelated to CAUTI, most of which were also unrelated to the device

Among the whole study population (n = 1000), 192 patients reported 283 adverse events (AEs). Most of the AEs were attributable to CAUTI (e.g. suprapubic tenderness, costovertebral tenderness; Fig. 6). The percentage of patients with at least one AE was significantly lower in the NMA-coated BIP Foley Catheter group than in the control group (21.6% vs. 48.4%; *p* = 0.001). Most AEs were mild, and there were no serious AEs related to the study devices or study-specific procedures.

### Hospital stay

The mean duration of hospitalization was 6.5 days in the BIP Foley Catheter group and 6.3 days in the control group (*p* = not significant; Table 1).

## Discussion

This randomized, multi-center, prospective clinical study demonstrated a significant reduction of CAUTI in patients receiving the NMA-coated BIP Foley Catheter versus an uncoated control catheter. A ~ 60% reduction in bacteriuria translated to a ~ 70% reduction in CAUTI incidence over the whole study period. The cumulative incidence of CAUTI was significantly lower in the BIP Foley Catheter group between 3 and 30 days, showing that the device is effective in both short- and medium-term use. Importantly, this study did not reveal any safety concerns with the BIP Foley Catheter.

There were no significant differences in the baseline characteristics of patients in the two arms of the study, except for the proportion of females being significantly lower in the BIP Foley Catheter arm than in the control arm (31% vs 40%). Female gender is a known risk factor for CAUTI, but the subgroup analysis of our data demonstrated no interaction between the effectiveness of the BIP Foley Catheter and gender (Fig. 4c). The BIP Foley Catheter produced numerical reductions in the incidence of CAUTI in all analyzed subgroups (based on age and body mass index [BMI] as well as gender) and, in all except two of the subgroups (patients of old age [> 60 years] and those with low BMI [< 20]), these reductions were statistically significant. This suggests that the anti-infective effect of the BIP Foley Catheter is not restricted to specific subgroups of patients, and that the robustness of our results was not reduced by the small gender-related difference between the two study arms.

The NMA coating of the BIP Foley Catheter is applied to the inner and outer surface of the catheter shaft and to the balloon and tip of the device, thereby minimizing the potential for bacterial colonization and CAUTI. A recent case study showed no significant release of metal into the urine, meaning that the device is non-toxic and does not facilitate the development of resistance in bacteria [24]. These are important considerations in patients requiring catheterization for a long duration. The NMA coating is postulated to have a galvanic mechanism of action which disturbs and prevents microbial adhesion to the surface. The results presented in Fig. 5 suggest that this is effective in reducing urinary levels of a wide spectrum of microbes including gram( −) and gram( +) bacteria, fungi, and the most abundant uropathogenic species.

To date, the present study is the largest randomized, multi-center study of an NMA-coated catheter to be performed using an internationally accepted definition of CAUTI. The results are consistent with previous studies showing positive outcomes with NMA-coated catheters, with reductions in the incidence of CAUTI between 35 and 90%, depending on the patient cohort, clinical setting and definition of CAUTI [11, 18–21, 24, 25]. However, some of the earlier studies should be interpreted with a degree of caution because they were not well-powered randomized controlled trials. One large, randomized study by Pickard et al. was performed to compare a noble metal alloy-coated latex catheter with a nitrofural-impregnated silicone catheter and a standard polytetrafluoroethylene (PTFE) coated latex catheter in patients requiring short-term catheterization (≤ 14 days) [26]. There were no significant between-group differences in the incidence of symptomatic CAUTI. However, the mean catheterization time of 2 days may have been too short for anti-infective effects to become apparent, and many patients would not have met the requirement for catheterization time ≥ 2 days specified by the CDC within their definition of CAUTI. Also, as described by Akre et al., the pragmatic methodology of this study (e.g. follow-up time for CAUTI surveillance was up to 6 weeks) increased the risk of both false-positive and false-negative CAUTI results [27].

Prevention of HAI is considered a global priority, with several initiatives from national and international organizations such as CDC and WHO. A recent systematic review and meta-analysis by Schreiber et al. [28] reported that HAI reductions of 35–55% can be achieved with multifaceted interventions. In one study, the implementation of multiple measures for preventing CAUTI (“5-S CAUTI bundle”, an approach that included education, catheter stabilization, correct positioning of the collection bag and daily evaluation) resulted in a statistically significant 80% reduction in CAUTI rate [29]. This is in the same range as the reduction achieved in the present study with the NMA-coated BIP Foley Catheter (69%). On a practical level, implementation of the 5-S CAUTI bundle strategy would require considerable time and resource (e.g. for staff training and ensuring maintenance of the required practices). Combined use of an NMA-coated catheter and measures included in the 5-S CAUTI bundle could be considered, as the different measures are likely to complement one another.

One limitation of our study was the short follow-up period after removal of the catheter; this meant that data on antibiotic use for the treatment of CAUTI were not sufficient for analysis. Further limitations included the lack of blinding of the healthcare personnel involved in the clinical assessment of CAUTI, and the potential for patient-reported symptoms to be affected by subjective interpretations. On the other hand, performance of the study in India was a strength because this is a country with very high levels of antimicrobial resistance and problems with infectious diseases. Background infection rates are much higher than in the developed world, increasing the chance of observing the effects of infection prevention measures. Another strength of the study was the definition of CAUTI according to CDC criteria (2014): this ensured objective diagnosis and minimized the risk of bias.

## Conclusions

This study confirms the efficacy of the NMA-coated BIP Foley Catheter in reducing the incidence of CAUTI and bacteriuria. The device was found to be well tolerated and no safety concerns were apparent. These data support use of the NMA-coated BIP Foley Catheter in clinical practice as a means of preventing CAUTI.

## Data Availability

The datasets used and/or analyzed during the current study are available from the corresponding author on reasonable request.

## Abbreviations

AE: Adverse event
BIP: Bactiguard infection protection
BMI: Body mass index
CAUTI: Catheter-associated urinary tract infections
CDC: Centers for Disease Control and Prevention
CI: Confidence interval
CTRI: Clinical Trials Registry-India
eCRF: Electronic case report form
HAI: Healthcare-associated infections
ICU: Intensive care unit
ISO: International organization for standardization
NMA: Noble metal alloy
OR: Odds ratio
RR: Relative reduction
SAS: Statistical Analysis Software (SAS)
UTI: Urinary tract infection
